# Role of *GSPT1* and *GSPT2* polymorphisms in different outcomes upon Hepatitis B virus infection and prognosis to lamivudine therapy

**DOI:** 10.1042/BSR20181668

**Published:** 2019-03-29

**Authors:** Wenxuan Liu, Ning Ma, Xia Gao, Wencong Liu, Jinhai Jia, Longmei Tang, Man Li, Lei Yang, Tao Li, Lina Yan, Xiaolin Zhang, Fengxue Yu

**Affiliations:** 1 Department of Epidemiology and Statistics, School of Public Health, Hebei Medical University, Hebei Province Key Laboratory of Environment and Human Health, Shi Jiazhuang, China; 2 Department of Social Medicine and Health Care Management, School of Public Health, Hebei Medical University, Shi Jiazhuang, China; 3 Department of Ultrasonics, The First Affiliated Hospital of Hebei Medical University, Shi Jiazhuang, China; 4 Department of Outpatient Clinic, School of Public Health, Hebei Medical University, Shi Jiazhuang, China; 5 Department of Science and Technology, The Hebei Key Laboratory of Gastroenterology, The Second Hospital of Hebei Medical University, Shi Jiazhuang, China

**Keywords:** HBV infection, GSPT, lamivudine, polymorphisms

## Abstract

***Purpose.*** *ERF3*, having been found expressing differently in liver tissues in our previous work, including *eRF3a* and *eRF3b*, which are structural homologs named *GSPT1* and *GSPT2*. Recent studies have indicated that *eRF3b* involved in the development and proliferation of HepG2 cell, and *eRF3a* may be associated with tumor susceptibility. Based on this, we tested the effects of *GSPT1* and *GSPT2* single-nucleotide polymorphisms for all major Hepatitis B virus (HBV) outcomes and lamivudine (LAM) treatment in Han Chinese. ***Method.*** A total of 1649 samples were enrolled, and peripheral blood samples were collected in the present study. The single-nucleotide polymorphisms in the *GSPT1* and *GSPT2* region were genotyped using MALDI-TOF MS. ***Results.*** Our study demonstrated there was no obvious relevance of either *GSPT1*-rs33635 or *GSPT2*-rs974285 polymorphisms with HBV susceptibility, spontaneous recovery, and development of HBV-related diseases. However, we showed for the first time to our knowledge that *GSPT1*-rs33635C was a predictor for LAM therapy (viral response: odds ratio (OR) = 2.436, *P*=0.022; biochemical response: OR = 3.328, *P*=1.73 × 10^−4^). ***Conclusions.*** These findings might provide potential implications for therapeutic guidance.

## Introduction

Hepatitis B virus (HBV) infection has become a major global health problem with a total of approximately 350 million chronic carriers distributed worldwide [1]. Data from epidemiological investigations showed a higher infection rate in Asia and Africa, with 5.3–12% of the hepatitis B surface antigen (HBsAg) carriers being Chinese, 8% living in Thailand, and 10% in Africa [2]. The clinical outcome of the infection varies widely and may include natural clearance (NC), chronic hepatitis, HBV-related liver cirrhosis (LC), or hepatocellular carcinoma (HCC) [3,4].

The complicated pathogenesis of HBV infection and the prognosis upon therapeutic intervention is attributable to the virus itself, immune state, genetic background of host, and environmental condition [5]. Genetic epidemiology indicates that amongst all the contributory factors, host genetic components play a vital role [6]. Experimental results have revealed a series of genes potentially associated with HBV-related diseases and the outcome of treatment: including *TNF, PDCD1, CTLA-4, TGF-α*, and *CXCL-10* were associated with persistent HBV infection [7–12], our previous study also indicated that *HLA, PAP*L, *IL10RB*, and *DEPDC*5 loci were associated with both HBV-related diseases and HBV clearance, and *HLA* allele polymorphism had relevance with the outcomes of lamivudine (LAM) treatment [13,14].

Moreover, in our previous work, two differential molecular peptides had been found by MALDI-TOF MS, one of which was identified as ‘*GSPT2* eukaryotic peptide chain release factor GTP-binding subunit *eRF3*’. In mammals, the two genes encoding *eRF3* (*eRF3a* and *eRF3b*) are structural homologs, have been identified and named *GSPT1* and *GSPT2*. Our research showed that *eRF3a/GSPT1* and *eRF3b/GSPT2* were positively expressed in liver tissues. Compared with normal controls, the relative expression of *GSPT2*/18s rRNA was higher in chronic hepatitis B (CHB) patients than in patients with either LC or HCC [15]. So we considered *eRF3b/GSPT2* is probably associated with the development of HBV-related liver disease. Our further study has indicated that *eRF3b* promotes viability, proliferation, and cycle progression of HepG2 cell [15]. Meanwhile, in recent years, studies have found that *eRF3a* may be associated with tumor susceptibility [16–18]. But it is not understood whether *GSPT1* and *GSPT*2 polymorphism is relevant to HBV infection and prognosis upon therapeutic intervention in Chinese people.

The present study was designed to explore the association of *GSPT1* rs33635 and *GSPT2* rs974285 polymorphisms with HBV susceptibility, NC, and the development of HBV-associated diseases. In addition, further investigation was carried out for testing the relevance of the *GSPT1* and *GSPT2* polymorphisms with prognosis after therapy with LAM.

## Materials and methods

### Patients

Patients were recruited from the Fifth Hospital of Shijiazhuang, The First and Second Affiliated Hospital of Hebei Medical University from January 2010 to January 2012. A total of 1649 samples were genotyped and analyzed in the present study. The samples included 507 cases of healthy controls, 350 cases of NC, 484 cases of chronic HBV infection (225 cases of CHB and 259 cases of LC) and 308 cases of HBV-associated HCC. Amongst them, 354 cases used LAM for the first time. The first goal of the study, which examined HBV carriers and healthy controls, was to confirm the possible association of the selected SNPs with HBV susceptibility. The second goal was to assess the possible association of the selected SNPs with the capacity to achieve spontaneous control of HBV infection once HBV infection became persistent in HBV carriers and patients exhibiting NC of HBV. The third goal of the study, which in patients with different HBV infection status, was to define the association of selected SNPs with progression of HBV-related liver disease. The last goal was to estimate the relevance of the selected SNPs with the outcome of LAM therapy in viral or biochemical response group and nonresponse group.

To be identified as healthy, individuals had to meet the following clinical criteria: (i) no record of hepatitis B vaccine history; (ii) negative for HBsAg, hepatitis B e antibody (anti-HBe), hepatitis B core antibody (anti-HBc) and other HBV biomarkers; (iii) normal blood routine examination and biochemical parameters; (iv) no history of endocrine disorders, cardiovascular events, or liver- and kidney-associated diseases. HBV NC was defined based on the following criteria: positive for hepatitis B surface antibody (anti-HBs) and anti-HBc definite absence of HBsAg accompanied by normal liver function and no history of HBV vaccination. CHB was divided into two groups according to HBeAg expression: HBeAg positive and HBeAg-negative CHB. Cirrhosis was diagnosed based on clinical, biochemical, radiological, or histological findings. HCC diagnosis was confirmed by a pathological examination and/or α-fetoprotein elevation (>400 ng/ml) combined with imaging. The sequence of tests used to diagnose HCC depended on the size of the lesion: (i) those with a focal hepatic mass >2 cm underwent imaging wherein characteristic contrast enhancement features on the arterial phase with venous washout on an MRI or CT could be demonstrated and (ii) those with a focal hepatic mass with atypical imaging findings, or a focal hepatic mass detected in a noncirrhotic liver, underwent a liver biopsy [19,20].

The requirements for LAM treatment were definite diagnosis of CHB or LC with a serum HBV DNA level of >5 log copies/ml, a serum alanine aminotransferase (ALT) level two times higher than the upper limit of normal ALT range and LAM (100 mg/day) use for the first time. Those receiving LAM treatment were divided into two groups according to the virological response (DNA load <3 log copies/ml or reduction in DNA load >2 log copies/ml): viral response group and viral nonresponse group. Two other groups were obtained depending on the biochemical response (ALT concentration within normal levels): biochemical response group and biochemical nonresponse group [21,22].

The information on all subjects under investigation included the following: baseline information (gender, age, history of smoking, drinking, and HBV vaccine etc.), historical disease background (condition and course of disease, with or without other disorders, family history), results of biochemical and serological testing (HBV markers, quantity of HBV DNA, ALT and AFP concentrations). The precise description of smoking and alcohol drinking was as follows: individuals who smoked one cigarette per day for over 1 year were defined as smokers, and those who consumed one or more alcohol drinks a week for over 6 months were considered alcohol drinkers. Informed consent was obtained from each subject, and the Institutional Review Board of Human Research of Hebei Medical University approved the study protocol.

### Genotyping

Blood samples were collected in tubes containing ethylenediaminetetra-acetic acid (EDTA). A genomic DNA Purification Kit purchased from Promega was used for genomic DNA extraction and TOF MS technology from the American SEQUENOM company for all sample SNP genotyping. Primer design for the two SNP alleles was performed by the Hua Da Gene company with the aid of MassARRAY Assay Design 4.0 Software (Sequenom Inc., San Diego, CA, U.S.A.). All reactions, including PCR amplification, shrimp-alkaline-phosphatase treatment and base extension, were performed in 384-well plates (ABI, Carlsbad, CA, U.S.A.). Following quality control plan was employed for data reliability: (i) Negative control setting; (ii) carrying out quality control criterion indoors; and (iii) genotyping in both replication sample sets had been performed in 5% of the total samples.

### Statistical analysis

Continuous variables of normal distribution were expressed as means ± S.D. ($\bar{x}\pm s$) and data of abnormal distribution expressed by median and interquartile range, *M (Q)*. Categorical variables are presented as frequencies. Comparisons between continuous data were carried out using Student’s *t* test and Wilcoxon’s test. The associations between categorical variables were evaluated using a Pearson’s chi-squared test. The chi-squared G test (goodness of fit) was employed to verify whether the proportions of the GSPT1-rs33635C and GSPT2-rs974285C polymorphism genotypes were distributed in accordance with the Hardy–Weinberg equation (Supplementary Table S1). Nonconditioned logistic regression was utilized to adjust involved factors such as age, gender, smoking, alcohol drinking etc., and odds ratio (OR) and 95% confidence intervals (95% CIs) were calculated. The most common genotype in the healthy controls was considered the reference genotype. To evaluate the effect of the genotype containing the SNP variant, we ran analyses assuming codominant, dominant, and recessive models. In a model in which codominant effects of the variant (V) and wild-type (W) alleles were assumed, the genotypes W/W, W/V, and V/V were coded as 0, 1, and 2, respectively. When a dominant effect was assumed, genotype W/W was coded as 0, whereas W/V and V/V were both coded as 1. Similarly, W/W and W/V were both scored as 0, and V/V was scored as 1 in a model that assumed a recessive effect. A *P*-value of 0.05 was considered significant in all analyses, and all *P*-values were two-sided. α value is adjusted by Bonferroni’s correction for multiple comparisons. All analysis mentioned above were performed using SPSS version 16.0 (SPSS Inc., Chicago, IL, U.S.A.).

## Results

### Demographic and basic characteristics of the subjects

The demographic parameters and the alcohol and tobacco consumption of all participants are listed in Table 1. Above information and clinical characteristics of viral response subjects and biochemical response subjects are presented in Supplementary Tables S2 and S3.

**Table 1:** Demographic and etiological characteristics of study subjects

Characteristics		Age	Gender (%)		Smokers (%)		Drinkers (%)		Log_HBV DNA_	HbeAg (+/−)	
		(mean ± S.D.)	Male	Female	Yes	No	Yes	No	(mean ± S.D.)	Yes	No
HBV susceptibility	CIB (*n*=792)	49.45 ± 13.48	535 (67.7)	256 (32.3)	355 (44.8)	437 (55.2)	395 (49.9)	397 (50.1)	6.56 ± 2.32	295 (37.2)	497 (62.8)
	Healthy (*n*=507)	49.29 ± 14.61	297 (58.6)	210 (41.4)	141 (27.8)	366 (72.2)	171 (33.7)	336 (66.3)	-	0 (0.0)	507 (100.0)
	*P*	0.845	0.001		7.43 × 10^−10^		1.03 × 10^−8^			-	
	OR (95% CI)		1.478 (1.173–1.862)		2.109 (1.660–2.679)		1.955 (1.552–2.463)			-	
HBV clearance	HBV NC (*n*=350)	50.32 ± 14.95	216 (61.7)	134 (38.3)	101 (28.9)	249 (71.1)	119 (34.0)	231 (66.0)	-	0 (0.0)	350 (100.0)
	CIB (*n*=792)	49.45 ± 13.48	535 (67.7)	256 (32.4)	355 (44.8)	437 (55.2)	395 (49.9)	397 (50.1)	6.56 ± 2.32	295 (37.2)	497 (62.8)
	*P*	0.330	0.052		3.79 × 10^−7^		6.66 × 10^−7^			-	
	OR (95% CI)		1.296 (0.998, 1.685)		2.003 (1.528, 2.624)		1.931 (1.487–2.508)			-	
CHB→LC	LC (*n*=259)	51.11 ± 11.12	148 (57.1)	111 (42.9)	84 (32.4)	175 (67.6)	101 (39.0)	158 (61.0)	6.30 ± 2.31	96 (37.1)	163 (62.9)
	CHB (*n*=225)	38.61 ± 14.37	142 (63.1)	83 (36.9)	95 (42.2)	130 (57.8)	103 (45.8)	122 (54.2)	6.23 ± 2.18	101 (44.9)	124 (55.1)
	*P*	2.33 × 10^−23^	0.181		0.026		0.132		0.733	0.081	
	OR (95% CI)		0.779 (0.541, 1.124)		0.657 (0.453, 0.952)		0.757 (0.527, 1.088)			0.723 (0.502, 1.041)	
LC+CHB→HCC	HCC (*n*=308)	55.96 ± 9.06	245 (79.8)	62 (20.2)	176 (57.1)	132 (42.9)	191 (62.0)	117 (38.0)	7.02 ± 2.36	98 (31.8)	210 (69.2)
	LC+CHB (*n*=484)	45.30 ± 14.17	290 (59.9)	194 (40.1)	179 (37.0)	305 63.0)	204 (42.1)	280 (57.9)	6.27 ± 2.25	197 (40.7)	287 (59.3)
	*P*	9.52 × 10^−35^	5.68 × 10^−9^		2.68 × 10^−8^		5.02 × 10^−8^		7.20 × 10^−6^	0.012	
	OR (95% CI)		2.643 (1.895, 3.687)		2.272 (1.697, 3.041)		2.241 (1.673, 3.002)			0.680 (0.503, 0.918)	

Abbreviation: CIB, HBV infection.

### Association of GSPT1-rs33635C and GSPT2-rs974285C polymorphism with HBV-related diseases

Tables 2 and 3 show the distribution of genotypes of *GSPT1*-rs33635 and *GSPT2*-rs974285 in all groups, we found no statistical association between the genotypes and the presence of HBV infection, HBV NC, LC development from CHB, HCC development from CHB and LC in three models. The unconditional logistic regression analysis, with adjustments for age, sex, smoking drinking, HBV DNA, and HbeAg (+/−), also showed no statistically significant relevance between *GSPT1*-rs33635C or *GSPT2*-rs974285C and HBV-related diseases.

**Table 2:** Association of *GSPT1* variant rs33635 with HBV outcomes

	Cases, *n* (%)			Controls, *n* (%)			Codominant		Dominant		Recessive	
	TT	CT	CC	TT	CT	CC	OR (95% CI)	*P*	OR (95% CI)	*P*	OR (95% CI)	*P*
HBV susceptibility	CIB (*n*=792)			Healthy (*n*=503)								
	318	350	124	180	243	80	1.140 (0.810, 1.604)^1^	0.452	0.831 (0.659, 1.047)	0.115	0.982 (0.723, 1.333)	0.905
	(40.2)	(44.2)	(15.7)	(35.8)	(48.3)	(15.9)	0.890 (0.639, 1.240)^1^	0.479^2^	0.804 (0.635, 1.019)^1^	0.071^2^	1.004 (0.735, 1.372)^1^	0.979^2^
HBV NC	CIB (*n*=792)			NC (*n*=348)								
	318	350	124	143	158	47	0.992 (0.752, 1.309)^1^	0.957	1.040 (0.805, 1.344)	0.766	1.189 (0.828, 1.708)	0.349
	(40.2)	(44.2)	(15.7)	(41.1)	(45.4)	(13.5)	1.231 (0.829, 1.829)^1^	0.302^2^	1.047 (0.807, 1.358)^1^	0.731^2^	1.236 (0.856, 1.786)^1^	0.258^2^
CHB→LC	LC (*n*=259)			CHB (*n*=225)								
	105	107	47	95	95	35	1.007 (0.635, 1.703)^1^	0.978	1.072 (0.746, 1.540)	0.708	1.204 (0.745, 1.944)	0.448
	(40.5)	(41.3)	(18.1)	(42.2)	(42.2)	(15.6)	1.016 (0.608, 1.834)^1^	0.955^2^	1.028 (0.749, 1.615)^1^	0.888^2^	1.017 (0.639, 1.592)^1^	0.942^2^
CHB+LC→ HCC	HCC (*n*=308)			CHB+LC (*n*=484)								
	118	148	42	200	202	82	1.206 (0.847, 1.685)^1^	0.286	1.134 (0.846, 1.519)	0.399	0.774 (0.517, 1.158)	0.212
	(38.3)	(48.1)	(13.6)	(41.3)	(41.7)	(16.9)	0.816 (0.527, 1.261)^1^	0.361v	1.102 (0.784, 1.301)^1^	0.452^2^	0.680 (0.451, 1.125)^1^	0.098^2^

Abbreviations: CIB, HBV infection.

^1^OR and OR 95% CI adjusted for age, sex, smoking status, drinking status, HBV DNA, HbeAg (+/−). ^2^α value is adjusted by Bonferroni’s correction and statistically significant results (*P*<0.025).

**Table 3:** Association of *GSPT*2 variant rs974285 with HBV outcomes

	Cases, *n* (%)			Controls, *n* (%)			Codominant		Dominant		Recessive	
	TT	CT	CC	TT	CT	CC	OR (95% CI)	*P*	OR (95% CI)	*P*	OR (95% CI)	*P*
HBV susceptibility	CIB (*n*=769)			Healthy (*n*=483)								
	29	59	681	19	34	430	1.113 (0.709, 1.7481)^1^	0.641^2^	1.048 (0.730, 1.505)	0.798	0.957 (0.531, 1.727)	0.884
	(3.8)	(7.7)	(88.6)	(3.9)	(7.0)	(89.0)	1.016 (0.554, 1.864)^1^	0.958^b^	1.079 (0.744, 1.564)^1^	0.689^2^	1.008 (0.550, 1.847)^1^	0.979^2^
HBV NC	CIB (*n*=769)			NC (*n*=322)								
	29	59	681	14	27	281	0.884 (0.545, 1.434)^1^	0.618^2^	0.886 (0.596, 1.316)	0.547	0.862 (0.449, 1.654)	0.655
	(3.8)	(7.7)	(88.6)	(4.3)	(8.4)	(87.3)	0.755 (0.465, 1.7431)^1^	0.755^2^	0.890 (0.596, 1.329)^1^	0.568^2^	0.909 (0.470, 1.759)^1^	0.778^2^
CHB→LC	LC (*n*=248)			CHB (*n*=213)								
	10	21	217	8	14	191	1.113 (0.651, 2.067)^1^	0.716^2^	1.240 (0.694, 2.215)	0.466	1.077 (0.417, 2.773)	0.879
	(4.0)	(8.5)	(87.5)	(3.8)	(6.6)	(89.7)	1.130 (0.487, 2.815)^1^	0.785^2^	1.192 (0.693, 1.910)^1^	0.497^2^	1.107 (0.467, 3.150)^1^	0.835^2^
CHB+LC→ HCC	HCC (*n*=308)			CHB+LC (*n*=461)								
	11	24	273	18	35	408	0.958 (0.534, 1.628)^1^	0.880^2^	0.987 (0.627, 1.553)	0.955	0.912 (0.424, 1.958)	0.812
	(3.6)	(7.8)	(88.6)	(3.9)	(7.6)	(88.5)	1.009 (0.480, 2.364)^1^	0.982^2^	1.006 (0.602, 1.588)^1^	0.981^2^	0.993 (0.516, 2.368)^1^	0.986^2^

Abbreviations: CIB, HBV infection.

^1^OR and OR 95% CI adjusted for age, sex, smoking status, drinking status, HBV DNA, HbeAg (+/−).

^2^α value is adjusted by Bonferroni’s correction and statistically significant results (*P*<0.025).

### Association of GSPT1-rs33635C and GSPT2-rs974285C polymorphism with viral and biochemical responses

Our study suggested that the *GSPT1*-rs33635C allele polymorphism had relevance with the viral response to LAM therapy. In the recessive genetic models, individuals carrying the rs33635C allele had a higher chance of responding to LAM in relation to viral load (OR: 2.624, 95% CI: 1.191–5.778, *P*=0.014). After adjustment for gender, age, smoking, alcohol, HBV DNA, and HbeAg (+/−), there was an association of rs33635C with the viral response in the codominant genetic models (rs33635CC compared with rs33635TT, adjusted OR: 2.436, 95% CI: 1.159–5.328, *P*=0.044). In the recessive genetic models, rs33635CC provided increased chances of responding to LAM therapy in relation to the viral response (adjusted OR: 2.607, 95% CI: 1.149–5.715, *P*=0.038). In addition, the rs33635 polymorphism showed similar relevance to the biochemical response: in the recessive genetic models, rs33635CC carriers displayed 3.947 higher than rs33635CT+TT carriers (95% CI: 1.999–7.794, *P*=3.09 × 10^−5^). There was still relevance between rs33635 polymorphism and biochemical response after adjustment for gender, age, smoking, alcohol, HBV DNA, and HbeAg (+/−): in the codominant genetic model carrying rs33635CC alleles had an increased possibility of a biochemical response (adjusted OR: 3.328, 95% CI: 1.841–6.830, *P*=0.001) with an adjusted OR of 3.762 (95% CI: 1.848–7.369, *P*=3.47 × 10^−4^) in the recessive genetic models (Table 4).

**Table 4:** Association of *GSPT1* rs33635 and *GSPT*2 rs974285 with LAM therapy

	Cases, *n* (%)			Controls, *n* (%)			Codominant		Dominant		Recessive	
	TT	CT	CC	TT	CT	CC	OR (95% CI)	*P*	OR (95% CI)	*P*	OR (95% CI)	*P*
Viral response rs33635	107	99	46	48	46	8	0.962 (0.528, 1.532)^1^	0.887^2^	1.139 (0.718, 1.809)	0.580	2.624 (1.191, 5.778)	0.014
	(42.5)	(39.3)	(18.3)	(47.1)	(45.1)	(7.8)	2.436 (1.159, 5.328)^1^	0.022^2^	1.097 (0.638, 1.749)^1^	0.719^2^	2.607 (1.149, 5.715)^1^	0.019^2^
Viral response rs974285	10	14	210	10	2	90	3.285 (0.842, 15.848)^1^	0.109^2^	0.857 (0.411, 1.789)	0.681	0.411 (0.165, 1.020)	0.049
	(4.2)	(5.7)	(90.1)	(6.8)	(4.3)	(88.8)	0.567 (0.268, 1.084)^1^	0.111^2^	0.904 (0.458, 1.826)^1^	0.775^2^	0.395 (0.158, 0.940)^1^	0.041^2^
Biochemical response rs33635	76	65	42	79	80	12	0.916 (0.541, 1.375)^1^	0.712^2^	1.155 (0.758, 1.758)	0.503	3.947 (1.999, 7.794)	3.09×10^−5^
	(41.5)	(35.5)	(23.0)	(46.2)	(56.8)	(7.0)	3.328 (1.841, 6.830)^1^	1.73×10^−4 (2)^	1.180 (0.728, 1.815)^1^	0.478^2^	3.762 (1.848, 7.369)^1^	1.74×10^−4 (2)^
Biochemical response rs974285	8	11	153	12	5	147	2.481 (0.835, 7.610)^1^	0.107^2^	1.074 (0.537, 2.146)	0.840	0.618 (0.246, 1.553)	0.302
	(4.7)	(6.4)	(89.0)	(7.3)	(3.0)	(89.6)	0.693 (0.248, 1.561)^1^	0.435^2^	1.112 (0.580, 2.215)^1^	0.756^2^	0.586 (0.231, 1.511)^1^	0.955^2^

^1^OR and OR 95% CI adjusted for age, sex, smoking status, drinking status, HBV DNA, HbeAg (+/−).

^2^α value is adjusted by Bonferroni’s correction and statistically significant results (*P*<0.025).

In the dominant and codominant genetic models, *GSPT2* rs974285 polymorphism has no relevance with either viral response or biochemical response, even when gender, age, smoking, alcohol, HBV DNA, and HbeAg (+/−) were adjusted.

## Discussion

The present study for the first time demonstrated that there was no obvious relevance of the *GSPT1*-rs33635C and *GSPT2*-rs974285C polymorphisms with HBV susceptibility, spontaneous recovery, and the development of HBV-related diseases. However, we found significant association between the *GSPT1*-rs33635C allele polymorphism and the viral and biochemical responses to LAM therapy.

Eukaryotic release factors are encoded by two distinct genes [23,24], *eRF3a/GSPT1* and *eRF3b/GSPT2*, which are located on human chromosome 16 and X, respectively. Chauvin et al. [25] indicated that eRF3a is the major factor that acts in translational termination in mammals, and eRF3b can substitute for *eRF3a* in this function. *ERF3a* and *eRF3b* share 87% identity for both mRNA and protein sequences, which differ in their N-terminal domains. *ERF3* has been reported to be involved in translational termination [26], cell-cycle regulation [27], and other cellular processes such as cytoskeleton organization and tumorigenesis [28].

In recent years, studies have shown that *eRF3a* may be associated with tumor. Malta-Vacas et al. [17] reported that *eRF3a/GSPT112* - GGC alleles can increase the susceptibility of breast cancer. In addition, Brito et al. [16] indicated that the *eRF3a/GSPT1* may be associated with gastric cancer susceptibility, and the analysis also revealed that different expression of *eRF3a/GSPT1* may be related to different histological types of gastric cancer [28]. In our previous work, we found that *eRF3a/GSPT1* and *eRF3b/GSPT2* were highly expressed in liver tissues. *GSPT2* mRNA was, however differentially expressed in CHB and LC/HCC. This differentially expressed protein could change the cell cycle and influence the phosphorylation status of 4E-BP1 on Ser^65^ in HepG2 [15]. Although their roles in the pathologic mechanisms of action in HBV-related diseases are still unknown, they can be used as potential biomarkers in diagnosis and prognosis. Through the Hapmap retrieval, we received two SNPS in *GSPT1* and *GSPT2* gene loci (rs33635 and rs974285), but found no statistical association between both *GSPT1*-rs33635 and *GSPT2*-rs974285 loci and the susceptibility of HBV infection, HBV NC, LC development from CHB, HCC development from CHB and LC.

LAM is the earliest oral antiviral drug, which has been widely used to treat HBV infection for its good security. However, recent studies have found that long-term use of LAM can induce serious drug resistance, which was ascribed to be HBV virus variation in the current views [29,30], and whether the antiviral efficacy is associated with genetic factors is not clear. Therefore, the study further investigated the association between *GSPT1*-rs33635, *GSPT2*-rs97428 two SNPs and LAM treatment outcome. The present study found that rs33635 had relevance with the viral response, as well as biochemical response to LAM therapy, the individuals carrying rs33635CC had a higher chance of responding to LAM in relation to viral load and biochemical level. However, only under the recessive model, rs974285 revealed a weak association with DNA response (OR = 0.381, 95% CI = 0.149–0.976, *P*=0.044). Considering the proportion of rs974285 polymorphism genotypes were not distributed in accordance with the Hardy–Weinberg equation, so we have no reason to consider that there were any relationship between either viral response or biochemical response and rs974285.

Recent years, individualized antivirus treatment are put forward to HBV-related diseases in order to improve therapy effect. In this context, high response rates are particularly important for choosing drugs, and the individualized therapy has been focussed on to predict the outcome of treatment from genetic level. In view of this, our team made the case–control study in Chinese people for 3 years, found *HLA-DQ* rs2856718 and rs9275572 two polymorphic loci had relevance with the prognosis of LAM treatment [10], as well as *GSPT*1 rs33635. Further, we will verify the prediction effect of *HLA-DQ* and *GSPT1* SNPs on LAM therapy, and to explore the interaction between gene and gene, genes and environment, to build the model of polygenetic and environmental factors to predict the effect of LAM therapy.

Taken together, our study suggests that *GSPT* 1oci were candidate susceptibility regions that had marker SNPs for outcomes of LAM treatment in Han Chinese. Our study supports the notion that carriage of the *GSPT1*-rs33635C might be critical for virus elimination and play an important role in LAM treatment. These findings might provide potential implications for therapeutic guidance. We will continue to explore the relationship between investigated gene polymorphisms and the corresponding protein. In addition, further study should focus on finding biomarkers of predicting the outcome of LAM therapy through the epidemiological studies and molecular mechanism research, as to provide theoretical basis for individualized treatment of HBV-related diseases.

## Supporting information

**Supplementary Table 1:** Hardy-Weinberg test of 2 SNPs in healthy and spontaneously recovered subjects

**Supplementary Table 2:** Baseline clinical characteristics of viral response subjects

## Abbreviations

4E-BPI: 4E-binding protein 1
95%CI: 95% confidence interval
AFP: automated fibre placement
ALT: alanine aminotransferase
anti-HBc: hepatitis B core antibody
CHB: chronic hepatitis B
CTLA-4: cytotoxic T lymphocyte antigen 4
CXCL-10: CXC chemokine ligand-10
ERF3: eukaryote polypeptide-chain release factor 3
HBsAg: hepatitis B surface antigen
HBV: hepatitis B virus
HCC: hepatocellular carcinoma
HLA: human leukocyte antigen
IL10RB: interleukin-10 receptor B
LAM: lamivudine
LC: liver cirrhosis
NC: natural clearance
OR: odds ratio
PAPL: purple acid phosphatase-like
PCDC1: programmed cell death 1
SNP: single-nucleotide polymorphism
TGF: transforming growth factor
TNF: tumor necrosis factor
