# Supplementary material for: Role of GSPT1 and GSPT2 polymorphisms in different outcomes upon Hepatitis B virus infection and prognosis to lamivudine therapy
Source: Biosci Rep. 2019 Mar 29;39(3):BSR20181668. doi: 10.1042/BSR20181668 (PMC6438872; doi:10.1042/BSR20181668)
Supplement: Supplementary file 1 [file bsr-39-bsr20181668_Supp1.pdf]

Supplementary Table 1 Hardy-Weinberg test of 2 SNPs in healthy and spontaneously recovered subjects

SNP	Genotype	ObsNum.	MAF	χ^2	*P*
	TT	323			
rs33635	CT	401	0.385	0.020	0.889
	CC	127			
	TT	711			
rs974285	CT	61	0.079	184.355	＜0.001
	CC	33			

Supplementary Table 2 Baseline clinical characteristics of viral response subjects

characteristics	Chronic hepatitis B		*P*	OR(95%CI)
	viral Response	viral Non-response		
Age（$\bar{\chi} \pm s$）	50.86 ± 11.03	52.25 ± 10.75	0.273	
Gender(Male/Female)	160/92	83/19	0.001	0.398(0.227,0.697)
Smokers(Yes/No)	81/171	48/54	0.008	0.533(0.333,0.853)
Drinkers(Yes/No)	105/147	55/47	0.036	0.610(0.384,0.970)
HbeAg(+/-)	129/123	55/47	0.641	0.896(0.565,1.421)
HBV DNA (log pg/ml) ,*M(Q)*	7.67（0.47）	7.69（0.44）	0.345	
ALT (IU/L) ,*M(Q)*	279.50（188.00）	317.50（220.00）	0.096	
AST (IU/L) ,*M(Q)*	357.50（272.00）	326.00（230.00）	0.614	

Supplementary Table 3 Baseline clinical characteristics of biochemical response subjects

characteristics	Chronic hepatitis B		*P*	OR(95%CI)
	Biochemical Response	Biochemical Non-response		
Age（$\bar{\chi} \pm s$）	52.36 ± 10.83	50.09 ± 10.98	0.051	
Gender(Male/Female)	111/72	132/39	0.001	0.455(0.286,0.725)
Smokers(Yes/No)	54/129	75/96	0.005	0.536(0.346,0.831)
Drinkers(Yes/No)	70/113	90/81	0.007	0.558(0.365,0.851)
HbeAg(+/-)	78/105	80/91	0.431	0.845(0.556,1.286)
HBV DNA (log pg/ml) ,*M(Q)*	7.66（0.55）	7.69（0.48）	0.438	

ALT (IU/L) ,*M(Q)*	279.00（174.00）	306.00（223.00）	0.186
AST (IU/L) ,*M(Q)*	331.00（278.00）	361.50（239.00）	0.796
